# Editorial Notes

**Published:** 1909-09

**Authors:** 


### Editorial Notes.

**British Medical Association, 1909.**

For the following notes on the Belfast Meeting we have to thank Dr. George Parker, the representative of the Bristol Division.

At the annual meeting of the British Medical Association, held this year at Belfast, about 1,000 members were present in spite of the voyage and the distance from the English centres of population. Several subjects of special interest to Bristol men were under discussion. Belfast is of much the same size as Bristol, and it is instructive to notice how she is meeting similar difficulties to those which confront us. At the present time she is just opening a new University of her own in place of the old Queen's College. The Medical School, dating from 1820, has suffered from the changes of the Irish University system during the last fifty years, but remains a vigorous body with some 270 students.

The two general hospitals have together about as many beds as our two institutions, but our Convalescent Home is larger than anything in Belfast. They have, however, a great number of special hospitals for children, maternity, skin, throat and ophthalmic cases, and in addition the huge hospital of the Poor-Law Union, with 1,500 beds, besides the fever hospitals and lunatic asylum. All of these are utilised for clinical instruction by special arrangements, so that they claim to have more than 3,500 beds available for students. Thus, for example, on one day in the week senior students are taken round the great Poor-Law Hospital. The largest general hospital has been rebuilt recently in an open area distant from the docks. It consists of seventeen pavilions, all on the same floor, and is beautifully equipped for its work. Finally, sanatoria for consumptives provide about 350 beds.

Sir William Whitla told us of the difficulties they have met with in laying down the course of medical studies in the new University, more particularly with respect to the ever-growing demands of the preliminary subjects. Under the new regulations the latter are to be concluded in the first year, and anatomy and physiology in the second, so that three full years must be devoted to clinical work.

A new feature in the meeting this year was the gathering of the Medical Libraries' Association, which we owe to the exertions of Professor Walker Hall. A large audience assembled at an early hour to hear a most fascinating address by Professor Osler in support of the movement, the subject being "Medical Libraries as a Means of Post-Graduate Study." This is to be published shortly, and is sure to be read with universal interest. In the exhibition of rare and curious medical works arranged by the Association, the splendid volumes shown by the kindness of the Bristol Corporation and the Bristol Medical Library were much admired. Another exhibition of a different kind was the wonderful collection of fractures, the work of Alexander Gordon, the celebrated Professor of Surgery in Belfast for nearly forty years. Indeed, there were so many places and institutions to be seen that time was wanting to make a proper examination.

The treatment of tuberculosis was the most prominent subject at the meeting. The Viceroy and Lady Aberdeen spent a large part of the week at Belfast listening to lectures and presiding over social gatherings. Lady Aberdeen's crusade against consumption in Ireland is one of the most useful movements of the time, and at the ladies' entertainment in the Opera House she gave a little address with limelight views of the work of her association. Belfast is especially proud that her distinguished professor, Henry MacCormac, in 1855 advocated the open-air treatment of phthisis. His treatise on the subject was exhibited with a long series of others, showing the growth of the idea down to the present-day hand-books used by Lady Aberdeen's society. The city too has been the first to erect a huge sanatorium for Poor-Law cases at White Abbey, some seven miles out. Two hundred and sixty cases were there at the time of my visit, the advanced cases in large wards and the others in small pavilions. Many of the patients were sleeping entirely in the open air under sheds, and all of them taking a plentiful diet under ordinary sanatorium rules. At Forster Green and the Throne sanatoria for consumptives a hundred more beds are provided for patients of other classes, while throughout Ireland Lady Aberdeen is endeavouring to get disused coastguard stations and other buildings for treating the sick in their own districts. Only last month Ireland put in force a a stringent Tuberculosis Prevention Act, so that there is reason to think that in a short time Ireland will be far ahead of this country in organising a campaign for stamping out the disease. With respect to this campaign it was a striking fact that in Belfast, with a population the same as that of Bristol, there were, as we have said, no less than 350 beds provided for consumptives besides private sanatoria for the wealthy classes. One of the points in Philip's address was the extreme frequency of first attacks of tuberculosis in children, and the importance of its recognition by every available test. It added to the interest that Calmette himself read a paper in another room on his ophthalmic reaction. After this Osler jokingly expressed the hope that he and Calmette might not be isolated from the world if they were found to give a positive result from past illness.

The Representative Meeting was fortunate in having a comfortable hall with good acoustic properties, and got through a large amount of business with despatch. The greatest regret was expressed at the retirement of the chairman, Dr. J. A Macdonald of Taunton, who has most ably managed the representative meetings through a stormy and important period. It is hoped that before the next session the Charter will be an actual fact, and that the Association will be possessed of a new income from the valuable shops on the ground floor of its new building.

Important resolutions were passed as to whole-time medical officers of health, the medical inspection and treatment of school children, a special class of consultants, the rules of district nursing societies, and various hospital difficulties.

**The University of Bristol.**

We heartily congratulate the Medical School, the profession, and the public that the University of Bristol is now an accomplished fact, and will begin its career on October 1st. A meeting of the Court, whose constitution has been made widely representative of Bristol and the West of England, met early in July, and elected the Council, in which is vested the management of the general business of the University. Mr. Lewis Fry, who has done so much to promote the success of the University movement, was elected chairman. The Council received with much regret the resignation of the post of Vice-Chancellor by Professor Lloyd Morgan. He resigns it in order to be able to devote more time to his work in Psychology, the science in which he has justly earned high distinction. The Council and Senate both passed resolutions expressing their deep appreciation of his invaluable services to University College, Bristol, during so many years. After consultation with the Senate, the Council then proceeded to elect Sir Isambard Owen as Vice-Chancellor. Sir Isambard comes to us from Armstrong College, Newcastle-on-Tyne, where he has proved himself a most efficient administrator as Principal of that College. We think that this appointment will be of great value to the University, and it is especially fortunate for the Medical School that the new Vice-Chancellor's long experience in medical affairs as a member of the Council of the Royal College of Physicians and elsewhere will enable him to direct wisely the changes in its organisation, which will be necessary in order to convert it into the Faculty of Medicine of the new University. At a subsequent meeting Dr. J. Michell Clarke was appointed Pro-Vice-Chancellor. With regard to finance, it should be known that the University, although it has enough money to make a start, yet urgently requires further funds, for it begins its career as the poorest of the provincial Universities that have been recently founded. We trust that by the excellence of its work, and by the important place that it will occupy in the educational system of the country, it will attract to its coffers further large contributions from those who are generously disposed towards higher education in the West of

England. There is no better mode of helping the University at present than the endowment of Professorial Chairs for the advancement of research. We confidently hope that from small beginnings the University may enter upon a long career of success which will ever add to the renown of our ancient city.

The remarks of Sir William Whitla, in his presidential address at the 77th meeting of the British Medical Association, at Belfast, are of especial interest to us in Bristol just now. We quote a small portion of his address :—

"The problem of higher education presents at all times to the thoughtful mind a fascinating study, but on its purely literary and scientific aspects the present is not the occasion to dwell. The birth of a university brings at once to the mind of the medical man the serious consideration of the examination standards by which the new portal to the profession is to be guarded, and also the still more vital subject of the efficiency of the training and teaching to be carried out under its direct supervision. To the layman the event is of still greater importance ; the medical student of to-day becomes the physician or surgeon of to-morrow, armed with his lifelong commission in the struggle for the relief of suffering and the prevention or cure of disease, and in his hands are often placed the issues of life and death. For these reasons every member of the community becomes interested in or involved more or less at some period of his existence in the efficiency of the institution which has presided over the training of his medical attendant. Hence no pains should be spared by the municipality or by the State to make this training as perfect as possible. Many people are apt to think, if they think at all, that the recognition of the medical man as a valuable public or national asset is an idea of modern growth, but you all remember that stirring episode in the *Iliad*, where Homer, 3,000 years ago, makes Idomeus exclaim, when commanding Nestor in the din of battle to fly to the relief of the wounded leech, Machaon, 'when trembling Greece for her physician feared' :

> Ascend thy chariot, haste with speed away ;
> A wise physician, skilled our wounds to heal,
> Is more than armies to the public weal

It behoves us as medical men periodically to review our position at regards the teaching and practical training of those desirous of joining our ranks, and upon an occasion like the present no more suitable subject could occupy our attention than a survey of the existing conditions of medical education, and a scrutiny of the requirements and modifications of these methods necessitated by the progressive march of scientific research as knowledge grows from more to more. I am confident that such a survey will also incidentally reveal evidence to satisfy the medical profession, as represented by this great Association, that our new university is fully alive to its serious responsibilities, and will be able to maintain the efficiency and to uphold the dignity of medicine in this portion of his Majesty's dominions."

**Medical Inspection of Schools.**

The first report of the Medical Inspectors of Schools to the Bristol Education Committee for the period ending December 31st, 1908, consists of twenty-nine printed pages and numerous statistical tables. The period covered by the report is something under four months, as the appointments of the five medical officers were only made on September 1st, and some preliminary work had to be done before the regular and systematic inspections were taken in hand. Before noticing the main features of the report in detail, we may say generally that it is a most lucid, concise and businesslike document, and reflects great credit on the medical men who are responsible for its production. The material to be dealt with was naturally very bulky, and exceedingly complex, and it has been presented with a completeness which leaves nothing to be desired, while the general style of the report will render it interesting reading even to those who are not especially versed in the subjects with which it deals. We regard this as a point of no small moment. The work of the medical inspectors of schools is a work of far-reaching national importance, and every means should be taken to enlist the interest of the public in the results they have obtained, by well-written and well-arranged reports such as the one now under review. An idea of the work done may be gathered from the opening paragraphs—188 visits, usually lasting two and a half hours, have been paid, each inspector devoting a total of seven and a half hours a week to the work; 3,081 children have been inspected, these being in most cases new admissions or else children who left school last July. 1,321 of these required medical attention, 68 being unfit to go to school. With the mechanism of the medical inspection, which is fully set forth in this report, the profession in general is not directly concerned; but the results obtained, both statistical and practical, are of considerable interest. Thus, in one district, 99 out of 729 children or nearly 13.6 per cent., had a family history of phthisis; and while contagious skin diseases were rare (only 42 cases in all), dirty and verminous heads altogether amounted to 400 out of 3,081, or nearly 13 per cent. Actual starvation and rickets are not considered common in Bristol. Malnutrition is not uncommon, at any rate in the poorer districts. The medical inspectors report that the provision of a free meal has already had a beneficial effect on the nutrition of the poorer children. As showing the relation of regular employment of the parents to the nutrition of the children, the tables given on p. 21 are distinctly interesting, though the figures are not large. In a school where the parents were mostly in receipt of regular wages the height and weight of the boys on leaving were not only greater than those of boys from a school in a poorer district where there is much casual labour (and this after allowing for difference in age), but compared favourably to the heights and weights given by the anthropometric standard and those of boys leaving Marlborough College.

As regards the results of the inspections, in cases requiring general medical treatment, in only 35.6 per cent. had no action been taken by the parents. A nearly identical proportion of children with eye defects had not been taken for treatment by the parents. The cases with dirty or verminous heads, and those with contagious skin diseases, were all either cured or much improved under treatment, with the exception of twelve children who had left school. These results seem very satisfactory, and, in fact, the inspectors appear quite content with what has been achieved. They note, however, that the case is otherwise with regard to defective condition of the teeth. In only 18.6 per cent. of these had any treatment been carried out or promised. The remainder of the report is concerned with the co-relation of the medical inspection of schools and the Public Health Service, and the methods of dealing with blind, deaf and otherwise defective children in special institutions, &c. We note that nothing is said in the report as to the treatment of cases of ordinary illness or slighter defects in the institutions and dispensaries of the city, a point in the working of the system to which considerable importance will be attached by members of the profession.

**Tabetic Ataxia.**

An article on this subject by Dr. Maurice Faure, Physician to the Institute of La Malou (Hérault, France), which appeared in our *Journal* in 1906, vol. xxiv, should have been followed by a second on this subject, giving the methods and technical processes which are employed in the work of re-education of the muscles. Our distinguished confrère has not yet been able to carry out his intention; but meanwhile he forwards us a note on the Argyll-Robertson sign in tabes, and one on the paraplegias of old people. These are as follows :—

I. *Argyll-Robertson's Sign in Tabes.*

Argyll-Robertson's sign has been considered sometimes as a symptom of exceptional importance in tabes, pretabes, and the cerebro-spinal manifestations of syphilis. This sign does not, however, seem really to have either the constancy or perhaps the exceptional importance that some people have attributed to it. Several authors have pointed out lately that it could be entirely absent, or exist in only one pupil (Babinski, Ballet, Cestan, Eichhorst, Treupel, Mantoux, Milian, Rochon-Duvigneaud, Heitz, etc.).

Argyll's sign is the persistency of the "iridian" reflex to distance, with its abolition to light. We have examined 200 tabetics from this special point of view: seventeen had not got Argyll-Robertson's sign. For this study we had chosen observations of typical tabetics, with coarse and genuine symptoms.

All had avowed commemoratives of syphilis, and this affection, as well as tabes, had been diagnosed previously by other medical men (Raymond, Ballet, Babinski, Brissaud, Burlureaux, Leredde, Nageotte (of Paris), Pitres, Bergonié, Régis, Abadie (of Bordeaux), Perrin (of Marseilles), Dubois (of Bern), etc.).

In one case Argyll-Robertson's sign only existed in one pupil; in another one it had existed, but had disappeared; in another one we found irregular pupils; in two cases we observed a paradoxical reflex to light (the pupil being enlarged when someone is projecting a luminous "bundle"); in one case the reflexes, which were normal on one side, had remained nearly normal on the other one; in four cases there was only slackening or decrease of the pupillary reflexes; in seven cases, at least, an examination of the pupils did not reveal in the least a near modification of the reflectivity.

In short, the perturbations of the "iridian" reflexes do exist in nearly all cases of tabes, but the so-called Argyll-Robertson's sign fails in about eight per cent. of the cases.

All this enables me to admit that Argyll-Robertson's sign remains, with the vesical troubles, the abolition of the patellar reflex, and the "lightning-like" pains, as the four cardinal signs of tabes, and that pupillary disturbances are always the most valuable symptom of the cerebro-spinal accidents of syphilis, particularly in tabes. *But one may have to diagnose tabes even when not having Argyll-Robertson's sign, and perhaps, let us say, without any pupillary disturbance.*

II. *Paraplegias of Old People.*

Paraplegias of old people have a complex origin. Some authors have mentioned arterio-sclerosis and atheroma (Demange, Pic, Bonnamour), gaps of the nervous substance (Demange, Marie, Ferrand, Reverchon, Crouzon, Lejonne, Lhermite), alterations of the motive spinal cells (G. Ballet and M. Faure), etc.

The variety of clinical signs is not less important than that of the lesions. It is possible to observe symptoms having a cerebral origin, others a cerebellar, a spinal, a neurotic or muscular one, etc. The point of departure of all these accidents seems to be the vascular sclerosis and the general disturbance of the circulation and cellular nutrition which result from it.

In considering the subject from the point of view of evolution, prognosis and treatment, it seemed to me that it might be of some interest to distinguish two categories of senile paraplegias.

(1) Some are real spasmodic paraplegias, with middle or trifling contractures, exaggeration of reflexes, muscular weakening and relative motor impotence. These paraplegias are amenable to the method of treatment by passive exercises, which I notified for the genuine spasmodic paraplegias (1903–6). Improvement is slow, incomplete, but certain and durable.

(2) Others are really false paraplegias. The patient, when walking, makes short steps, and seems hardly able to drag himself along, raising his foot the least possible, and stumbling against obstacles. But on examining him, one may state that the reflexes are little modified or not, that the passive movements are easy, that any contracture or notable retraction does not impede them, that the patient's voluntary resistance to the imparted movements denotes a more than sufficient strength, that at last ordered voluntary movements are made with precision and amplitude. Thus, after a training of a few days and methodical voluntary exercises, one can obtain a real re-education of the gait; and such patients who were dragging themselves along, making 10-in. steps, walked afterwards at a quick pace, making 2-ft. 6-in. steps. Thus, in these cases, there is only a perturbation in the automatic mechanism of the gait, and not a disturbance in the muscular strength, co-ordination, sensibility, motive, will, etc.

Thus this improvement is got by other means than that pointed out in the preceding category, and the difference is made still more sensible by the fact of its being quick, easy, but unstable and often transitory. We are seeing every year, since 1902, old people showing, moreover, other symptoms of cerebro-spinal "senility," who get cured every season of their paraplegia, but forget often their treatment and its result some time after.

The difference we have just stated does not result only from the facts we observed. Professor Grasset, during the debate upon the senile brain at the sixteenth congress of alienist and neurologist medical men (Lille, August, 1906), pointed out similar facts, and made the same distinction. It seems that his opinion has been strengthened by the facts and conclusions quoted by Mr. Leri, reporter, as well as by H. Meige (reporter of *Revue Neurologique*, p. 764, August 30th, 1906).

### Notes on Preparations for the Sick.

